# Latency-Aware Task Scheduling for IoT Applications Based on Artificial Intelligence with Partitioning in Small-Scale Fog Computing Environments

**DOI:** 10.3390/s22197326

**Published:** 2022-09-27

**Authors:** JongBeom Lim

**Affiliations:** Smart Contents Major, Division of ICT Convergence, Pyeongtaek University, 3825, Seodong-daero, Pyeongtaek-si 17869, Gyeonggi-do, Korea; jblim@ptu.ac.kr

**Keywords:** fog computing, artificial intelligence, task scheduling

## Abstract

The Internet of Things applications have become popular because of their lightweight nature and usefulness, which require low latency and response time. Hence, Internet of Things applications are deployed with the fog management layer (software) in closely located edge servers (hardware) as per the requirements. Due to their lightweight properties, Internet of Things applications do not consume many computing resources. Therefore, it is common that a small-scale data center can accommodate thousands of Internet of Things applications. However, in small-scale fog computing environments, task scheduling of applications is limited to offering low latency and response times. In this paper, we propose a latency-aware task scheduling method for Internet of Things applications based on artificial intelligence in small-scale fog computing environments. The core concept of the proposed task scheduling is to use artificial neural networks with partitioning capabilities. With the partitioning technique for artificial neural networks, multiple edge servers are able to learn and calculate hyperparameters in parallel, which reduces scheduling times and service level objectives. Performance evaluation with state-of-the-art studies shows the effectiveness and efficiency of the proposed task scheduling in small-scale fog computing environments while introducing negligible energy consumption.

## 1. Introduction

The Internet of Things and applications are part of a multi-billion-dollar industry [1]. The technical reasons for its popularity are that small and lightweight devices with various sensors (for temperature, humidity, pressure, accelerometers, gyroscope, gas, infrared ray, or optics) are able to perform both real-time applications (video/audio conferencing, online games, real-time operating systems and middleware, instant messaging applications, etc.) and general applications in cloud-enabled fog computing environments [2,3]. Note that in fog computing environments, Internet of Things device tasks are offloaded to nearby edge servers [4,5]. Due to their lightweight properties, Internet of Things applications do not require supercomputer-class computing resources. Instead, a small amount of PC-class computing resources are enough for edge servers [6,7].

Thanks to edge servers in cloud-enabled fog computing environments, latency and response time can greatly be reduced since the Internet of Things devices communicate with nearby edge servers, not the central cloud datacenter. However, for real-time applications of the Internet of Things, unawareness of the fog computing architecture can result in poor latency, response time, and service level objectives [8,9]. In other words, in order to benefit from cloud-enabled fog computing environments, efficient and effective resource and task orchestrations are indispensable. However, enhancing the application performance is tricky and hard to implement in small-scale fog computing environments since the margin for improvement is also small [10,11].

Recently, various techniques based on artificial intelligence for fog computing resource and task management have been proposed [12,13]. The state-of-the-art studies for fog resource and task management proved that adopting artificial intelligence and related techniques (including artificial neural networks and reinforcement learning) is promising [14,15]. However, the downside of the existing artificial intelligence-based resource and task management is that scheduling time and service level objectives are sub-optimal since it involves hyperparameter training of artificial neural networks [16]. Hence, there is room for improvement in latency and response time for the real-time and Internet of Things applications in small-scale fog computing environments (under ten hosts).

In this paper, we propose a latency-aware task-scheduling method for Internet of Things applications based on artificial intelligence in small-scale fog computing environments. The core concept of the proposed resource and task scheduling is to use artificial neural networks with partitioning capabilities. With the partitioning technique for artificial neural networks, multiple edge servers are able to learn and train hyperparameters in parallel, which reduces the scheduling time and service level objectives.

The main contributions of the paper can be described below:

1. We propose a latency-aware task scheduling method for Internet of Things applications based on artificial intelligence in small-scale fog computing environments.
2. We introduce a partitioning technique that allows us to perform hyperparameter tunings in parallel with multiple edge servers.
3. We design and implement the artificial neural network and partitioning method suitable for real-time and Internet of Things applications in small-scale fog computing environments.
4. We compare performance results with state-of-the-art studies to show the effectiveness and efficiency of the proposed latency-aware fog resource and task management scheme.

The rest of the paper can be summarized as follows. Section 2 describes the research motivation and background with existing studies based on nature-inspired optimization and artificial intelligence. Section 3 details our latency-aware fog resource and task management technique for Internet of Things applications, partition-based parallel hyperparameter training methods, and algorithms to reduce scheduling time. Section 4 shows extensive experimental results in comparison with state-of-the-art studies. Finally, we conclude the paper with future research directions in Section 5.

## 2. Related Work

Task scheduling in fog computing environments is a well-known problem, and there are many approaches to solving the problem. Examples are genetic algorithm [17], swarm intelligence [18,19], ant colony optimization [20], and evolutionary techniques [21], to name a few. These nature-inspired optimizations are among the approximated and meta-heuristic solutions [22]. In other words, they explore the search space and find the optimal parameters in non-deterministic and efficient ways.

For example, genetic algorithms use a selection process inspired by human and biological evolution. There are six steps for genetic algorithms: (1) initialization based on a random function, (2) selection of parents based on a fitness function, (3) crossover and reproducing, (4) mutation of offspring, (5) evaluation for offspring, and (6) merging offspring. Although genetic algorithms are able to find near-optimal solutions efficiently, it is hard to customize for specific requirements.

Methods based on artificial intelligence have been proposed to overcome the limitations of nature-inspired optimization algorithms. The deep Q-learning approach utilizes neural networks like deep learning to help agents to choose the next action [23,24]. As the name suggests, it uses the Q-value (expected reward) to make the decision. Since deep Q-learning does not need to create a model for environmental transitions, it is called a model-free algorithm.

Deep reinforcement learning is another method based on artificial intelligence. A subfield of machine learning, it is a combination of reinforcement learning and deep neural networks [25,26]. Deep reinforcement learning uses predefined or simulated states, actions, and rewards to determine the loss function, which offers trajectory planning. As a variation, GOBI [27,28] runs on top of feed-forward artificial neural networks and updates gradients with respect to the input. The core process of deep neural network-based approaches is a learning process. Since task scheduling based on big workloads takes a huge amount of time, an efficient technique is urgently needed.

In artificial neural network designs, training and hyperparameter tuning can be done by backpropagation [29]. Since deep neural networks have hundreds or thousands of hyperparameters to learn, training on a single machine is limited in meeting real-time requirements. To this end, we adopt a parallelism technique for fast neural network learning in fog computing environments. Broadly, there are two approaches to the parallelism of neural network learning: data parallelism and model parallelism [30].

In fact, there is no global memory in fog computing environments like traditional distributed systems, and both data and model parallelisms are not easily achievable. The straightforward way to achieve parallelism is to use data partitioning [31]. For the input (workloads) of artificial neural networks, it splits input data into several parts, and a compute node (host) is asked to perform training for a split. Specifically, there are sub-branches for data parallelism: sample-level partitioning and feature-level partitioning.

The sample-level partitioning is to split data based on shuffling or random sampling. In contrast, the feature-level partitioning splits data based on feature dimensions, which capture neural network characteristics better. However, it requires more tasks to split data evenly than the sample-level partitioning as the number of features is unpredictable.

In model parallelism [32], a requirement to resolve a large learning model is to partition it into several parts, and each partition can be processed in different hardware modules such as a CPU, GPU, tensor processing unit (TPU), or neural processing unit (NPU). Since a commodity data center and edge servers are assumed, we consider data parallelism in this paper. The details of the proposed sample-level partitioning of data parallelism are provided in the next section.

## 3. The Proposed Method

The proposed method is based on the partitioning technique of artificial neural network learning: hence, the partitioning technique is integrated into the proposed scheduling algorithms. In this section, we detail the proposed partitioning techniques and scheduling algorithms in each subsection.

### 3.1. Architecture and Partitioning Framework

**Figure 1:**
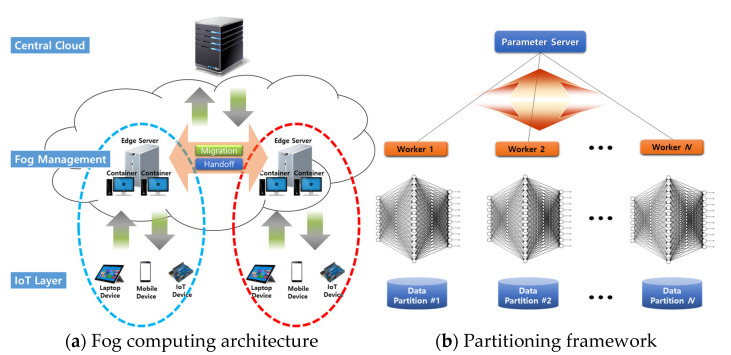
Fog computing architecture and partitioning framework.

Figure 1a shows our fog computing architecture composed of the central cloud server, fog management layer, and Internet of Things layer. The central cloud server is responsible for all operations within the architecture. The fog computing architecture offers edge servers for local processing and data storage to support real-time and Internet of Things applications. The fog management layer allows real-time and Internet of Things applications to reduce latency and use fewer bandwidth networks. Each edge server is able to provide a number of containers for computing capabilities. As far as migration costs are concerned, container migrations are more lightweight than virtual machine migrations.

Figure 1b depicts the partitioning framework. When the fog management layer asks edge servers to perform neural network training (backpropagation), one of the edge servers becomes a parameter server that distributes data sets to other edge servers and aggregates local models from them. Details of the partitioning technique are explained in the next subsection, but it is important to note that there is a tradeoff between the number of edge servers and the performance overheads. For the overheads, we perform a mini-test experiment in Section 4.

### 3.2. Partitioning Technique

The basic aim of using a partitioning technique is to allow multiple edge servers to perform hyperparameter training in parallel. Among various partitioning techniques, we adopt the sample-based-partitioning method, which is a comprehensive way to implement artificial neural networks. To this end, the sample-based partitioning uses a random approach for data shuffling to make sure each edge server has a subset of the global dataset for training.

The easiest way to implement the sample-based-partitioning method is to split the global dataset into the number of edge servers and allocate each split to the edge server without overlapping. However, when the global dataset is not a multiple of the number of edge servers, the sizes of the allocated data splits are not equal to each other. Thus, we assign a split to a randomly selected edge server, allowing each edge server to have one split for load balancing. Note that we assume that each edge server is capable of processing a split with available memory and storage.

A downside of using the sample-based-partitioning method is that the multiple uses of the random function can affect performance degradation. However, performance degradation due to the random function is limited since we consider a small-scale fog computing environment. Another potential weakness is that local training of edge servers may lead to the over-fitting problem. To avoid the problem, as we mentioned before, we use the data-shuffling technique at the beginning. In fact, the data-shuffling operations do not affect performance degradation because we do not consider large-scale environments.

The parameter server aggregates them from the edge servers to assemble the local models. More specifically, when an edge server finishes computing a local model, it informs the parameter server and sends its local model to the parameter server. When the parameter server completes the aggregation, the global model can be used for resource and task scheduling. It is worth noting that an edge server may fail and not inform the parameter server. In this case, the parameter server uses a timeout parameter to avoid indefinite waiting. The inherent property of artificial neural networks means the training problem with the loss of a local model would be minimal and would slow the training procedure down somewhat. We show how effective the sample-based-partitioning method is in Section 4 with real-world datasets.

### 3.3. Algorithms

To explain the overall algorithmic structure, we divide the algorithms into two parts: the parameter server and edge servers, in addition to the scheduling algorithm.

Algorithm 1 shows our partitioning algorithm for the parameter server. Note that we consider a single parameter server in artificial neural network learning. The input of the algorithm is a workload dataset, and the output is a global model (*Model_global*). There are a few steps for initialization before iterations. It initializes *Seed* based on the random function and retrieves information from edge servers (*Set_edge* and *Num_edge*). At this stage, there is no global model (*Model_global*); hence, this global variable is set to *null*. Since it is based on sample-level-partitioning, it calculates the total size of the dataset (*Total_size*) and configures the split size (*Split_size*).

The first iteration procedure (Lines 9–16) is to split data and allocate them to edge servers in the system. To assign a split to an edge server, it uses the make_split function with shuffling. The shuffle function is based on randomization, and the return value of the make_split function can be duplicated. To avoid duplicated splits, it performs the duplication_check function. When it is not duplicated, the split (*Split_i*) is assigned to *Edge_i* by the allocate_split function. Otherwise, it encounters the *continue* procedure (Line 13) and skips the rest of the iteration. The second iteration procedure is to aggregate local models for the parameter server. After assigning data splits to edge servers, it listens and waits until all the edge servers’ local models are aggregated. Whenever an edge server returns the local model to the parameter server, it updates the *Model_global* variable. Then it returns *Model_global*.
**Algorithm 1.** Partitioning Algorithm for Parameter Server.1: 2: 3: 4: 5: 6: 7: 8: 9: 10: 11: 12: 13: 14: 15: 16: 17: 18: 19: 20: 21:** Input:***Dataset* ** Output:***Model_global* ** Initialization:** *Seed* ← get_random(*CurrentTime*); *                           Set_edge* ← get_edgeservers(); *                           Num_edge* ← convert_to_num(*Set_Edge*) *                           Model_global* ← *null;* *                           Total_size* ← get_total_entry(*Dataset*); *                           Split_size* ← *Total_size*/*Num_edge*; ** for all**
*Edge_i* ∊ *Set_edge*
**do** // making data splits and allocate them to edge servers *            Split_i* ← make_split(*Dataset*, *Split_size*, shuffle(*Seed*, *Num_edge*)); *            Boolean* ← duplication_check(*Split_i*); *           *** if** *Boolean == false* **then** *                      * continue; *           *** end if** *           * allocate_split(*Edge_i*, *Split_i*); **end for** **for all**
*Edge_i* ∊ *Set_Edge*
**do** // aggregation of local models for data splits *            Local_model_i* ← retrieveLocalModel(*Edge_i*); *            Model_global* ← *Model_global* **∪** *Local_model_i*; **end for** **return**
*Model_global*

Algorithm 2 shows the sub-mode of the partitioning algorithm for edge servers. The input of the algorithm is *Split_i* from the parameter server, the pre-trained model (*Model_pre*), and *Workload*. The role of edge servers in the partitioning algorithm is simple. It trains the model for *Split_i* and returns the local model (*Local_model_i*) to the parameter server. Algorithm 3 shows the scheduling algorithm, whose input is *Model_global,* and the output is *SchedulingDecision*. The return value of the getModelFromPS function is the same as that of Algorithm 1. After receiving the *Model_global*, it converts to *SchedulingDecision*, which is interpretable for the scheduler. Then, it checks whether it needs task migrations. If the current scheduling decision is different from the previous scheduling decision for fog tasks, it schedules the tasks for migrations (Lines 5–7). Afterward, it saves the current scheduling decision (it can be the previous scheduling decision in the next cycle) for future use.
**Algorithm 2.** Partitioning Algorithm for Edge Server *i*.1: 2: 3: 4: 5: 6: 7: **Input:**
*Split_i* (can be allocated from the parameter server) *            Model_pre* (pre-trained model) *            Workload* **Output:**
*Local_model_i* *Split_i* ← getSplitFromPS(); *Local_model_i* ← train(*Workload*, *Model_pre*); // backpropagation **return**
*Local_model_i*;

**Algorithm 3.** Scheduling Algorithm.1: 2: 3: 4: 5: 6: 7: 8: 9: 10:**Input:** *Model_global* (can be retrieved from the parameter server) **Output:**
*SchedulingDecision* *Model_global* ← getModelFromPS(); v*SchedulingDecision* ← convertToDecision(*Model_global*); *Diff* ← searchForMigration(*SchedulingDecision*, *SchedulingDecision_previous*) **for each** *FogTask* ∊ *Diff* **do** *           *performMigration(*FogTask.source*, *FogTask.destination*); **end for** saveSchedule(*SchedulingDecision*); **return** *SchedulingDecision*;

## 4. Performance Evaluation

In this section, we perform extensive experiments and show the performance results in comparison with state-of-the-art studies: GA [33], DQL [34], DRL [35], and GOBI [27]. For the artificial neural network design, we use a feed-forward neural network model with two hidden layers, whose sizes are 128 and 64, respectively. The sigmoid activation function is used, and softplus and tanhshrink activation functions are optionally used for non-affine approximation. For workload datasets, we use BitBrain [36] and DeFog [37] datasets. For hosts (edge servers), we use a heterogeneous configuration with Azure B2 and B4 models. Azure B2 and B4 models use the same CPU (Intel Haswell 2.4 GHz E5-2673) with different RAM configurations: that is, 4 GB and 16 GB, respectively. For experimental results, we plot for 100 simulation intervals. Each interval is set to 300 s (5 min).

**Figure 2:**
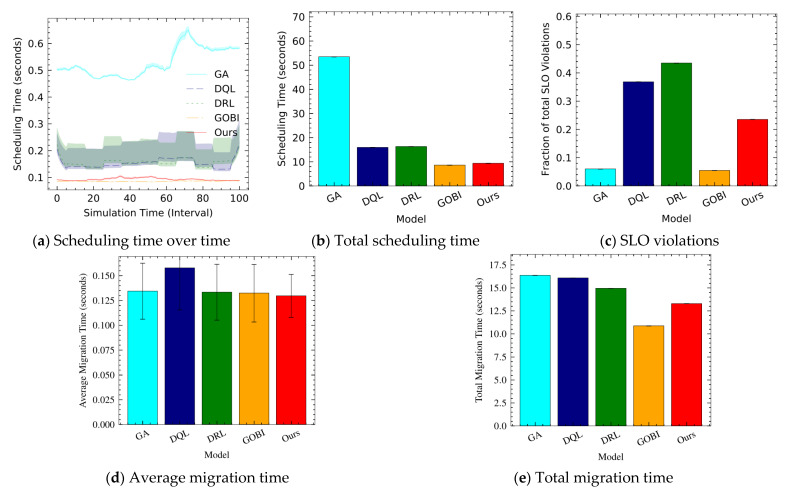
Mini-test experiments with six hosts.

To figure out the partitioning overhead, we configure a mini-test environment with six hosts (see Figure 2). Note that the other experiments are performed with ten hosts unless specified otherwise. In fact, in our partitioning framework, a parameter server is also responsible for local model processing. Hence, unbalancing exists between a parameter server and other edge servers.

Figure 2 shows scheduling time over time, total scheduling time, service level objective violations, average migration time, and total migration time. When the number of hosts is six, the scheduling time (Figure 2a,b) of GOBI and Ours is less than the other methods. However, Ours is slightly higher than GOBI for scheduling time. This difference stems from unbalancing caused by the partitioning technique. The unbalancing results in service level objective violations (Figure 2c). The service level objective violations of Ours are higher than GA and GOBI. Figure 2d,e shows average migration time, total migration time, and average wait time. For average migration time, Ours is the best, but for total migration time, Ours is higher than GOBI due to the total number of migrations.

Note that the service level objective is based on latency (response time), and the response time is the sum of scheduling time, wait time (container provisioning time), execution time, and migration time. However, the unbalancing problem can be mitigated when the number of hosts increases (e.g., to 10 or higher). In other words, local model processing time can be reduced when there are more hosts. Since we consider a small-scale environment, we leave ultra-scale performance (e.g., at 100 or higher) experiments as future work. Furthermore, the distance between a fog user and an edge server is tens to hundreds of meters, whereas the distance between edge servers can be hundreds of meters. In this regard, we can hypothesize that ten hosts are suitable for covering the Internet of Things devices.

**Figure 3:**
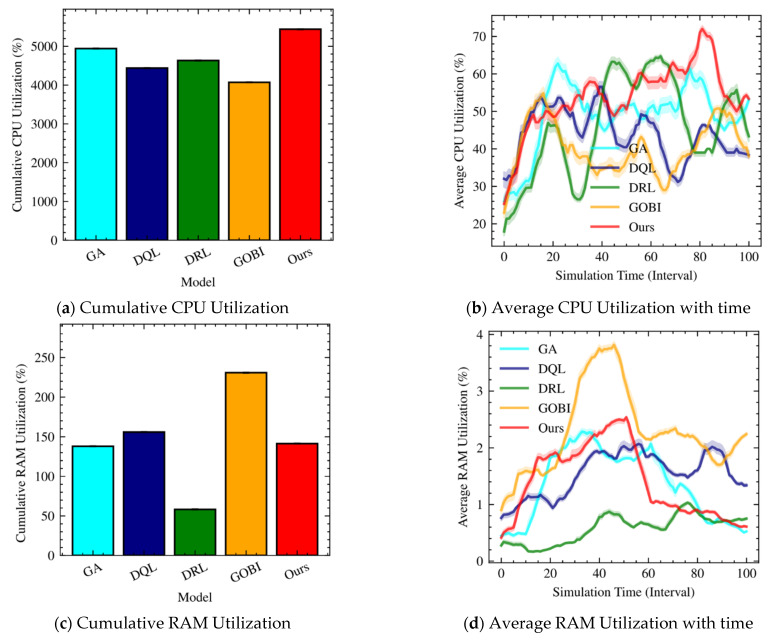
CPU and RAM utilization with ten hosts.

Figure 3 shows CPU and RAM utilization of containers for GA, DQL, DRL, GOBI, and Ours. For CPU utilization (Figure 3a,b), GA and Ours exhibit high CPU utilization relative to DQL, DRL, and GOBI. As far as server utilization is concerned in cloud computing environments, high CPU utilization is preferable for data centers’ costs and maintenance. In this regard, our approach meets the basic requirement while not exceeding 80% of CPU utilization. Note that maximum utilization of a CPU may result in high energy consumption due to boost (e.g., Turbo Boost and Turbo Core) and performance degradation due to race conditions.

Figure 3c,d shows the cumulative and average RAM utilization for containers. Because we consider fog computing environments based on containers, RAM utilization is a metric that is less because a single server supports a few thousand containers. Nevertheless, RAM utilization indirectly indicates the scheduling behavior. In other words, we can hypothesize that GOBI (high RAM utilization) performs fewer migrations while DRL performs larger migrations. Figure 3d depicts the average RAM utilization over time. As expected, GOBI fluctuates between 20 to 60 simulation times, while DRL exhibits little diversification. Our approach, however, gets stable after 60 simulation times.

**Figure 4:**
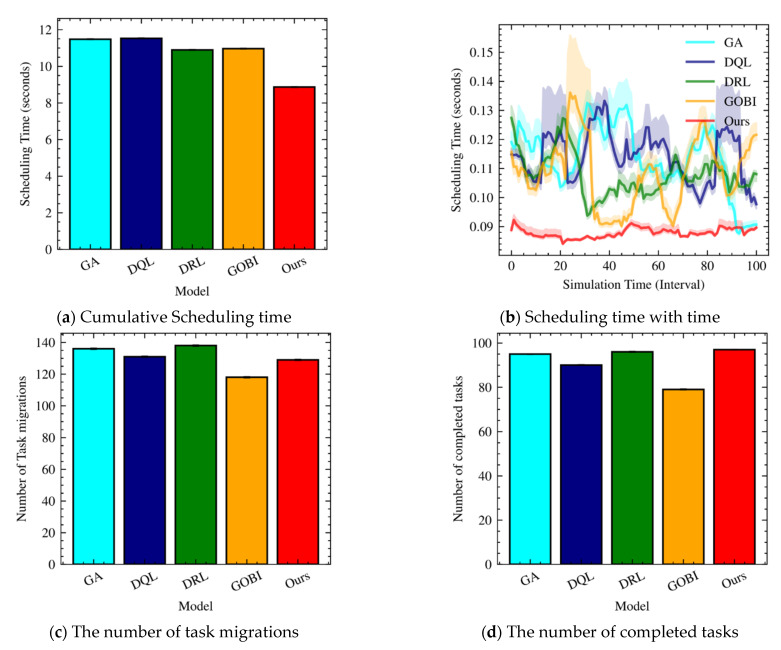
Scheduling time, the number of migrations, and the number of completed tasks with ten hosts.

Figure 4 shows scheduling time, the number of migrations, and the number of completed tasks for GA, DQL, DRL, GOBI, and our approach. When we orchestrate resource and task maintenance, scheduling time affects the response time and service level objectives. Since the Internet of Things applications have real-time properties, reducing the scheduling time is important for task scheduling. When we compare the scheduling time for the five techniques (GA, DQL, DRL, GOBI, and Ours), Ours is shorter than the other four. Specifically, the scheduling time of our technique is about 69.5% on average when compared to the other techniques.

Based on Figure 4b, we confirm that the scheduling time of our approach is both low and stable. Meanwhile, the other four techniques fluctuate much more than Ours, showing a high scheduling time. Figure 4c,d shows the number of task migrations and completed tasks. As we mentioned before, the highest task migrations can be found in DRL, and the lowest can be found in GOBI. On the other hand, our approach is positioned in the mid-range for the number of task migrations. In addition to the scheduling time, the number of completed tasks given time is an essential requirement for Internet of Things applications. When we see the number of completed tasks (Figure 4d), our approach recorded the highest number while avoiding over-scheduling for migrations. Even DRL performs more task migrations (Figure 4c). Ours is second-to-none regarding the number of completed tasks.

**Figure 5:**
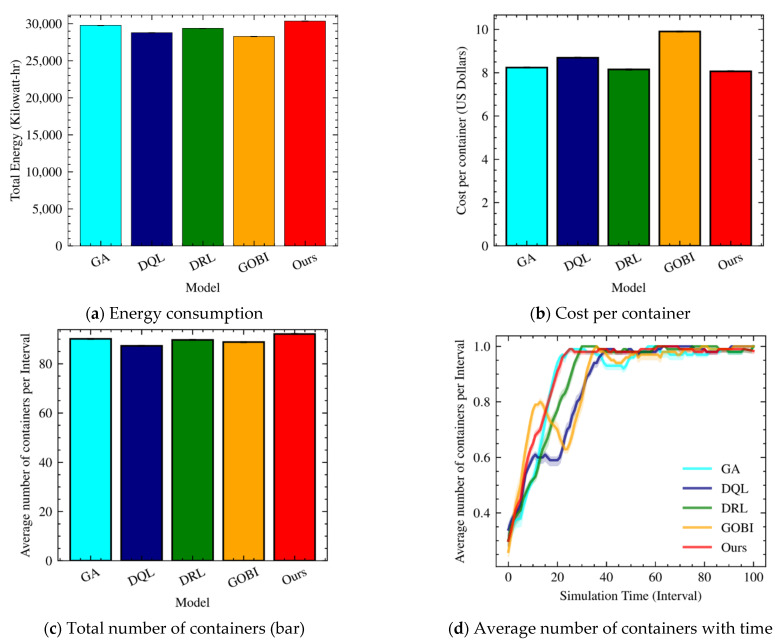
Energy consumption, cost per container, total number of containers, and average number of containers with ten hosts.

Figure 5 depicts energy consumption, cost per container (in USD), the total number of containers, and the average number of containers for GA, DQL, DRL, GOBI, and Ours. Note that the cost model is measured based on the cost list of Azure cloud machines by Microsoft for the South UK region. Surprisingly, the cost per hour of our approach is the lowest even though Ours consumes more energy consumption. This is because the basic cost model of the cloud computing service is an hourly-basis model. It signifies that our fog resource and task management in small-scale environments is expeditious.

More details about resource and task scheduling properties can be found in Figure 5c,d. Of the five comparison targets, Ours uses more containers: hence, it consumes more energy. When we look at the average number of containers (Figure 5d), DQL and GOBI fluctuate as the simulation time goes on. This results in more costs than others (Figure 5b). It also indicates that DQL and GOBI’s scheduling decisions are not accurate for the workload, while GA and Ours reach the top faster than others for the workload.

**Figure 6:**
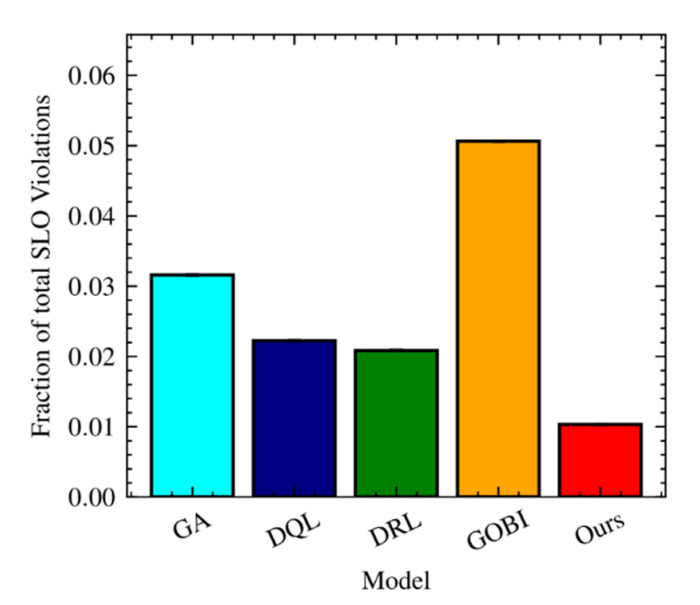
Service level objective violations with ten hosts.

Figure 6 shows service level objective violations for GA, DQL, DRL, GOBI, and Ours. Not surprisingly, our approach has the lowest service level objectives for Internet of Things applications. Note that for real-time and Internet of Things applications, the service level objectives are based on the response time. The major reason for this reduction of service level objective violations is contributed by enhanced scheduling time based on the proposed partitioning method. The highest number of violations can be found in GOBI due to wrong and mismatched scheduling decisions (Figure 3d). Compared with GOBI, the service level violations of the series Ours are about 1/5 (20%).

## 5. Conclusions

Speeding up the training of artificial neural networks is an essential research topic in deep learning. In this paper, we proposed the mapping from the scheduling problem of Internet of Things applications in fog computing environments to artificial neural networks and a parallelism mechanism for neural network training based on sample-level data partitioning. The proposed algorithms integrate the partitioning technique and task scheduling in small-scale fog computing environments. To figure out the performance of the proposed method, we compared the results with state-of-the-art studies. The experimental results show that our method is expeditious and suitable for real-time and Internet of Things applications. The proposed partitioning technique incurs bottlenecks in the aggregation stage, but the overhead is negligible in small-scale fog computing environments.

## Data Availability

Not applicable.
